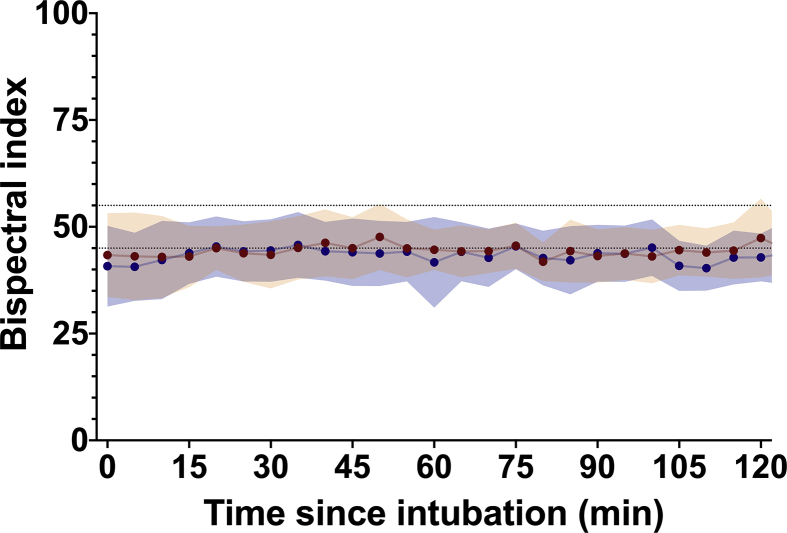# Reduced postoperative pain using Nociception Level-guided fentanyl dosing during sevoflurane anaesthesia: a randomised controlled trial

**DOI:** 10.1016/j.bja.2020.07.057

**Published:** 2020-09-17

**Authors:** Fleur Meijer, Maarten Honing, Tessa Roor, Samantha Toet, Paul Calis, Erik Olofsen, Chris Martini, Monique van Velzen, Leon Aarts, Marieke Niesters, Martijn Boon, Albert Dahan

**Affiliations:** 1 Department of Anesthesiology, Leiden University Medical Center, Leiden, Netherlands; 2 Department of Anesthesiology, Alrijne Hospital, Leiderdorp, Netherlands

**Keywords:** nociception, nociception level-guided anaesthesia, opioid, postoperative pain, stress hormones

## Abstract

**Background:**

The majority of postoperative patients report moderate to severe pain, possibly related to opioid underdosing or overdosing during surgery. Objective guidance of opioid dosing using the Nociception Level (NOL) index, a multiparameter artificial intelligence-driven index designed to monitor nociception during surgery, may lead to a more appropriate analgesic regimen, with effects beyond surgery. We tested whether NOL-guided opioid dosing during general anaesthesia results in less postoperative pain.

**Methods:**

In this two-centre RCT, 50 patients undergoing abdominal surgery under fentanyl/sevoflurane anaesthesia were randomised to NOL-guided fentanyl dosing or standard care in which fentanyl dosing was based on haemodynamics. The primary endpoint of the study was postoperative pain assessed in the PACU.

**Results:**

Median postoperative pain scores were 3.2 (inter-quartile range 1.3–4.3) and 4.8 (3.0–5.3) in NOL-guided and standard care groups, respectively (*P*=0.006). Postoperative morphine consumption (standard deviation) was 0.06 (0.07) mg kg^−1^ (NOL-guided group) and 0.09 (0.09) mg kg^−1^ (control group; *P*=0.204). During surgery, fentanyl dosing was not different between groups (NOL-guided group: 6.4 [4.2] μg kg^−1^*vs* standard care: 6.0 [2.2] μg kg^−1^, *P*=0.749), although the variation between patients was greater in the NOL-guided group (% coefficient of variation 66% in the NOL-guided group *vs* 37% in the standard care group).

**Conclusions:**

Despite absence of differences in fentanyl and morphine consumption during and after surgery, a 1.6-point improvement in postoperative pain scores was observed in the NOL-guided group. We attribute this to NOL-driven rather than BP- and HR-driven fentanyl dosing during anaesthesia.

**Clinical trial registration:**

www.trialregister.nl under identifier NL7845.

**Editor's key points**

• There are recognised challenges in optimising intraoperative analgesia with no direct measure of pain in anaesthetised patients.
• Integrating a wide range of relevant factors into an algorithm, as is used in the Nociception Level index, has shown some promise in guiding analgesia.
• Despite similar levels of opioid use and depth of anaesthesia, use of the NOL index to guide intraoperative fentanyl dosing resulted in reduced postoperative pain scores and a smaller increase in stress hormones (adrenocorticotropic hormone [ACTH] and cortisol) compared with standard care.
• These promising results require further study to understand the mechanisms of improved analgesia.

During surgery, the administration of opioid analgesics is directed at controlling nociception and accordingly preventing an adrenergic stress response evoked by surgical tissue injury.^1^ Correct opioid dosing during anaesthesia is important, not only to prevent underdosing, but similarly to prevent overdosing, which may cause hypotension and postoperative hyperalgesia (i.e. heightened postoperative pain responses).^2^, ^3^, ^4^ Nociception is not readily detected in an anaesthetised and relaxed patient, and in current clinical practice is qualified through measurement of BP and HR. In recent years, various monitors were developed to quantify nociception during anaesthesia.^5^ We and others showed that one such monitor, the Nociception Level (NOL) monitor (Medasense Biometrics Ltd, Ramat Gan, Israel), is able to reliably detect and quantify mild to intense noxious stimulation during anaesthesia and surgery and outperforms haemodynamic indices (BP, HR) in the ability to distinguish between noxious and non-noxious stimuli.^6^, ^7^, ^8^ The monitor makes use of an algorithm based on advanced statistical and machine learning technologies; it combines multiple autonomic signals (finger photoplethysmogram amplitude, skin conductance, HR, HR variability, and their time derivatives) into a single index, the NOL index.^9^ Machine learning was used to create the optimal algorithm to translate input (predictors) into output (NOL index) without the need of an *a priori* specified stochastic model. The index ranges from 0 (absence of nociception) to 100 (extreme nociception). The algorithm was validated in multiple studies.^2^^,^^6^, ^7^, ^8^, ^9^

A large proportion of postoperative patients report moderate to severe pain.^10^^,^^11^ Postoperative high pain scores have a negative impact on patient wellbeing and outcome, delay recovery, and are associated with prolonged use of opioids often also after discharge.^12^, ^13^, ^14^, ^15^ Therefore, interventions aimed at prevention of high postoperative pain will benefit the patient, and reduce prolonged opioid use and overall cost of care. One such intervention could be that opioid dosing based on nociception rather than on haemodynamic variables, such as BP and HR, improves postoperative pain scores by preventing underdosing or overdosing. We hypothesised that NOL-guided opioid dosing during surgery results in lower pain scores in the first 90 min stay in the PACU compared with standard clinical care in patients undergoing major abdominal surgery during sevoflurane/fentanyl anaesthesia. Additionally, we hypothesised that reduced pain scores are associated with lower concentrations of stress hormones (adrenocorticotropic hormone [ACTH], cortisol).

## Methods

### Ethics and patients

Patient recruitment started after the protocol was approved by the Leiden Human Ethics Committee in March 2019. All patients gave written informed consent before enrolment. The study was registered at trial register of the Dutch Cochrane Centre (www.trialregister.nl) under identifier 7845. The study took place from June 2019 to December 2019 at two medical centres in the Netherlands; the Leiden University Medical Centre, a tertiary referral centre, and the Alrijne Hospital, Leiderdorp, a secondary referral centre. The study was performed at both sites by anaesthesia caregivers who were trained in the use of the NOL. Standard care was comparable between the two sites. The study protocol is available from the authors at a.dahan@lumc.nl.

We recruited patients of ASA physical status 1–3, aged >17 yr, scheduled to undergo elective laparoscopic/robot-assisted abdominal surgery without epidural anaesthesia, local blocks, or infiltration. Exclusion criteria included inability to give informed consent, pregnancy/lactation, MAP at screening or on the day of surgery >160 mm Hg or <60 mm Hg, HR at screening or on the day of surgery >90 beats min^−1^ or <45 beats min^−1^, and any CNS-related disorder.

### Study design

#### Randomisation and allocation

Patients were enrolled by the investigators 1–2 weeks before surgery, so that the patient had ample time to consider participation. The study had a randomised, parallel, superiority design, with primary endpoint pain score in the first 90 min in the PACU. Randomisation to either NOL-guided analgesia or standard care was performed automatically within the electronic data capture system CASTOR (https://www.castoredc.com) in the operating room before induction of anaesthesia and could not be altered. Subjects and surgeons were not informed on the group assignment. In both allocation groups, the NOL monitor (PMD-200, Medasense Biometrics Ltd, Ramat Gan, Israel) was connected to the patient by a finger probe, placed on the left or right middle finger. In cases of NOL-guided analgesia the monitor screen was visible to the anaesthesia team and used to steer fentanyl administration. In cases of standard care, the nociception monitor screen was concealed and the NOL index was not available for guidance of fentanyl dosing, but index values were measured and collected on the hard disk of the monitor.

#### Common clinical care in the two centres

Before induction of anaesthesia, no sedatives or opioids were given. In both groups, subjects received an i.v. line, three-lead continuous ECG, BP cuff, TOF-Cuff (RGB Medical Devices, Madrid, Spain) for neuromuscular monitoring, and bispectral index (BIS) monitoring (Philips, Eindhoven, the Netherlands) for measurement of depth of anaesthesia. As mentioned, all subjects were connected to the nociception monitor by a finger probe, placed contralateral to the BP cuff. Additionally, a separate i.v. line was placed in the cubital vein for blood sampling used for stress hormone measurement. To measure hormone concentrations, 8 ml of blood was drawn at six time points: 10–30 min before induction, 1–2 min after intubation, 1–2 min after incision, at skin closure, 15 min into the PACU, and at discharge from the PACU. Both hormones were measured in plasma by an electrochemiluminescence immunoassay using the Cobas 8000 module e 602 (F. Hoffmann-La Roche Ltd, Basel, Switzerland) with detection limit 1–1200 ng L^−1^ and coefficient of variation <2%.^16^

Anaesthesia was induced with fentanyl (∼1.5 μg kg^−1^) followed by propofol (1–2 mg kg^−1^). After consciousness was lost as detected by BIS values <60, absence of eyelash reflex, and no response to verbal stimulation, a neuromuscular blocking agent, rocuronium 0.6 mg kg^−1^, was administered. After full relaxation, the trachea was intubated. During induction additional doses of fentanyl and propofol could be given. Next, sevoflurane inhalation started (target BIS values 45–55). The ventilator settings were such that the end-tidal *P*co_2_ was kept between 4 and 5 vol.%. Fluid administration was standardised to 5–6 ml min^−1^; additional fluids could be administered in cases of moderate to severe blood loss. Towards the end of surgery, the inspired sevoflurane concentration was tapered down. All patients with a residual neuromuscular block (train-of-four ratios <0.9) were reversed with sugammadex 2 mg kg^−1^ and extubated when neuromuscular function had normalised (train-of-four ratio ≥0.9), were breathing spontaneously, and were awake; in cases where neuromuscular function had normalised spontaneously at the end of surgery, no reversal agent was given. Each subject received preemptive treatment for postoperative pain: acetaminophen 1 g i.v. 30 min before surgery and an i.v. opioid, morphine (0.1–0.15 mg kg^−1^) or piritramide (0.06–0.10 mg kg^−1^), 45–60 min before the end of surgery.

In the PACU, additional i.v. doses of morphine or piritramide were given when pain scores were >4 on an 11-point numerical rating scale (NRS), ranging from 0 (no pain) to 10 (most intense pain imaginable): morphine 1–2 mg or piritramide 2–3 mg could be given at 5–10 min intervals until NRS<4. Pain scores were queried and opioids administered by fully study-blinded PACU nurses.

#### Fentanyl administration in the NOL-guided group

In the test group, fentanyl dosing was dependent on the NOL index, but BP and HR were additionally monitored and considered. In cases where the NOL index was >25 for at least 60 s, fentanyl 50–100 μg was administered in a patient >70 kg, and 25–50 μg in a patient of 70 kg or less.^26^ Higher or lower fentanyl doses could be given or opioids could be given below the NOL threshold if felt needed by the attending anaesthesiologist. After fentanyl was given, 5–10 min were allowed before the next evaluation took place. In cases where the index decreased below 25, no more fentanyl was administered. If the index was <25 and the MAP was <60 mm Hg, vasoactive medication (ephedrine, phenylephrine, norepinephrine), crystalloids, or both could be given. Irrespective of index value, when MAP was >100 mm Hg and not responsive to fentanyl, despite adequate and repeated dosing, and BIS values were <55, a vasodilator (nitroglycerine or natrium nitroprusside) or a continuous infusion of remifentanil could be given. If BIS values were >55, the inspired sevoflurane concentration was increased such that BIS decreased below 55, and the patient condition was reassessed.

#### Fentanyl administration in the standard care group

In this group, fentanyl dosing was dependent solely on haemodynamics (MAP, HR) as NOL index values were not available. If systolic BP was >140 mm Hg, tachycardia (>90 min^−1^), or both, fentanyl 50–100 μg was administered in a patient >70 kg, and 25–50 μg in a patient of 70 kg or less.^2^ If felt necessary, the attending anaesthesiologist could give higher or lower fentanyl doses or give fentanyl below these thresholds. After fentanyl was given, there were no time constraints when a next evaluation took place and consequently there were no restrictions in timing of fentanyl dosing. When MAP was >100 mm Hg and not responsive to fentanyl, despite adequate and repeated dosing, and BIS values were <55, a vasodilator or a continuous infusion of remifentanil could be given. In cases where high BP coincided with high BIS levels, the inspired sevoflurane concentration was first increased such that BIS decreased below 55, and the patient condition was reassessed. In cases of low BP (MAP<60 mm Hg) with concurrent low BIS values (<45), the sevoflurane concentration was lowered first and vasoactive medication could be given next. When vasoactive medication did not increase BP, crystalloids were infused.

### Data collection, primary and secondary/tertiary endpoints

All variables were collected in the electronic data capture system CASTOR. The primary endpoint of the study was the pain score, obtained from pain scorings at 15 min intervals for the first 90 min of PACU stay. Secondary endpoints reported here are: fentanyl consumption during anaesthesia (in cases of remifentanil administration, the dose was converted into fentanyl equivalents), sevoflurane concentrations, surgery and PACU times, time between reversal of neuromuscular block and extubation, morphine/piritramide consumption in the first 90 min of PACU stay (all piritramide doses were converted to morphine equivalents with piritramide 15 mg=morphine 10 mg), and occurrence of awareness. Tertiary endpoints were: BIS values during surgery, median pain score in the PACU, morphine/piritramide dosing during surgery, percentage of subjects with pain scores ≥6 or ≥8, stress hormone concentrations (ACTH and cortisol), and Aldrete scores in the PACU. The Aldrete score is a composite index with 0–2 points for limb movement, respiration, circulation, consciousness, and oxygen saturation, and reflects the level of recovery from anaesthesia.^17^ An Aldrete score of 9–10 indicates full recovery and PACU discharge readiness, although pain and other issues (nausea/vomiting, surgical complications, logistics) will delay discharge from the PACU.

### Statistical analyses

Since this study is the first to assess the effect of NOL-guided analgesia on postoperative pain scores, we based our sample size calculation on the assumption that a mean pain score difference of 2 NRS points on the NRS (with standard deviation=2) in the first 90 min of PACU stay, is clinically highly relevant.^18^^,^^19^ Twenty-five subjects per group give a power of >90% to detect such a difference.

The primary endpoint, the pain scores over time, was compared between treatments by generalised estimation equation with covariates sex and centre. Data analysis was performed in R (R Foundation for Statistical Computing, Vienna, Austria; https://www.R-project.org). Additionally, the median pain scores obtained over time were compared by the Mann–Whitney *U*-test. For secondary and tertiary endpoints, because statistical testing was exploratory rather than confirmatory, power calculations were not performed. Analyses of these data were by an independent two-tailed *t*-test, the Mann–Whitney test, or the χ^2^ test (GraphPad Prism 8.3.0 for Mac OS X, GraphPad Software, San Diego, CA, USA), depending on the type of data and data distribution. Because ACTH and cortisol hormone concentrations are correlated, the combined data set was analysed using a linear mixed model in the statistical package NONMEM.^20^ A *P*-value <0.05 was considered significant; for all endpoints, *post hoc* Bonferroni corrections for multiple comparisons were made to control for type one errors. Data are presented as mean (standard deviation) unless otherwise stated.

### Simulation study

To get an indication of the fentanyl concentration at the end of surgery, a simulation study was performed using the pharmacokinetic data set of Boom and colleagues,^21^ with time, dose, and weight as input to the model. Individual plasma concentration *vs* time profiles were estimated. The plasma concentrations at the end of surgery and the percentage change in concentration at 2 h after the end of surgery were compared between treatment groups by two-tailed *t*-tests.

## Results

A total of 75 patients were approached for participation in the study (Supplementary Fig. S1). Recruitment ended when the number of included subjects reached the calculated required sample size. Twenty-five were not enrolled or randomised because of a variety of reasons including refusal to participate (*n*=16), high BP upon screening (*n*=4), or logistic reasons (surgery was postponed, *n*=5). Fifty subjects were enrolled in the study, randomised, and allocated to treatment with 25 subjects in each treatment arm. Data from all 50 subjects were analysed with no subjects lost during follow-up. Subject characteristics are given in Table 1. None of the subjects reported pain before surgery or used any opioids before surgery. The two groups were well balanced with respect to patient characteristics and haemodynamic variables at screening with, for example, similar MAPs, 96 (9) and 98 (10) mm Hg in NOL-guided and control groups, respectively. The distribution of surgical procedures was similar between treatment groups: colon surgery 68%, gynaecology 28%, and urology 4%. There were no device-related safety events.

**Table 1:** Subject characteristics.

	Nociception Level-guided analgesia	Standard clinical care
*n*	25	25
M/F, *n*	10/15	12/13
ASA physical status 1/2/3, *n*	6/13/6	6/18/1
Age, yr	60 (47–67)	61 (43–71)
Weight, kg	82 (18)	80 (14)
Height, cm	175 (9)	175 (11)
BMI, kg m^−2^	26.9 (5.5)	26.4 (3.5)
MAP, mm Hg∗	96 (9)	98 (10)
HR, beats min^−1^∗	72 (13)	74 (11)
General surgery, *n*	15	19
Gynaecology, *n*	9	5
Urology, *n*	1	1

All values are represented as mean (standard deviation), median (inter-quartile range), or numbers (*n*).

ASA, American Society of Anesthesiologists; F, female; M, male.

∗ Values obtained in the preoperative screening clinic

### Pain in the PACU

**Fig. 1:**
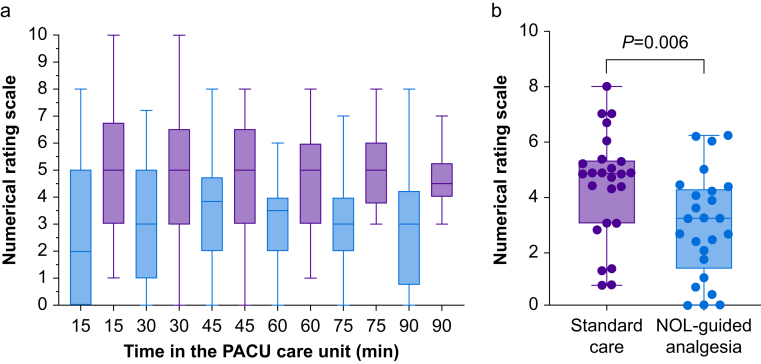
(a) Box plot of the pain scores at each of the measurement points in the PACU. Red boxes: standard clinical care; blue boxes Nociception Level (NOL)-guided analgesia. (b) Box plots of the median pain scores observed per subject in the PACU. Each symbol reflects the individual median pain scores during the subject stay in the PACU.

PACU pain scores over time are given in Figure 1a. Pain scores were consistently higher in subjects who received standard care compared with those who received fentanyl dosing based on the NOL index (generalised estimation equation: *P*=0.002, with no effect of covariates sex or centre). The median pain scores of individual subjects in the PACU were 3.2 (inter-quartile range 1.3–4.3) and 4.8 (3.0–5.3) in NOL and control groups, respectively (*P*=0.006, actual difference 1.6 with 95% confidence interval [CI] 0.5–2.7; Figure 1b). In the standard care group and NOL-guided group 15/25 and 8/25, respectively, had severe pain in the PACU (NRS≥6; χ^2^, *P*=0.047). No difference was observed in patients with intense pain (NRS≥8): control group 6/25 *vs* NOL-guided group 3/25 (*P*=0.270).

### Measurements made during surgery

**Fig. 2:**
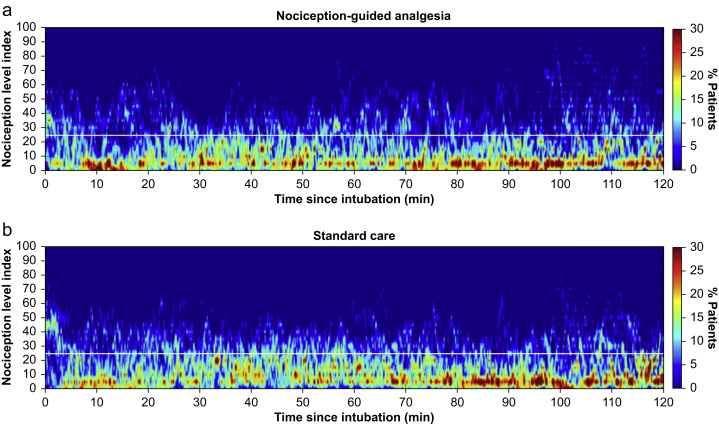
Fire plots of the Nociception Level (NOL) index values during anaesthesia in NOL-guided analgesia (a) and during standard care (b) patients. To guide the eye, one horizontal line is added to the graph representing a NOL index value of 25. The colours reflect the percentage of subjects at any 5-s time point and range from 0% (dark blue) to 30% (dark red).

Figure 2 gives the distribution of NOL values for the first 2 h of anaesthesia (fire plots of the 5-s output of the NOL device). Surgery, anaesthesia and recovery times, depth of anaesthesia (as measured by BIS; Supplementary Fig. S2), and sevoflurane concentrations were similar between NOL-guided and control patients (Table 2). The average expiratory sevoflurane concentration was 1.5–1.6%, which corresponds to an age-adjusted minimum alveolar concentration value of 0.9–0.95. The morphine and piritramide dose (in morphine equivalents) administered during surgery did not differ between treatment groups (Table 2). There was no difference in the use of vasoactive medication between groups. A total of 16 and 17 patients in the NOL-guided and control groups, respectively, received ephedrine, phenylephrine, or both. Four of these patients received an infusion with norepinephrine, two in each of the treatment groups. One patient in the intervention group received atropine 0.5 mg for bradycardia.

**Table 2:** Variables collected during and after surgery.

	Nociception Level-guided analgesia	Standard clinical care	Mean or actual difference (95% CI)	*P*-value
Anaesthesia time (min)	148 (67)	133 (45)	14 (−18 to 46)	0.377
Surgery time (min)	130 (63)	119 (44)	10 (−21 to 41)	0.512
End-tidal sevoflurane conc. (%)	1.6 (0.3)	1.5 (0.3)	0.06 (−0.1 to 0.2)	0.509
Mean bispectral index during surgery	44 (5)	44 (6)	0.05 (−3 to 3)	0.975
Cumulative fentanyl dose (μg kg^−1^)∗	6.4 (4.2)	6.0 (2.2)	0.35 (−1.93 to 2.654)	0.749
Morphine equivalents during surgery (mg kg^−1^)	0.13 (0.04)	0.14 (0.05)	−0.003 (−0.03 to 0.02)	0.812
Reversal time (min)†	9.5 (3.3)	9.3 (4.8)	0.2 (−3 to 4)	0.920
Median pain score in PACU	3.2 (1.3–4.3)	4.8 (3–5.3)	1.6 (0.5–2.7)	0.006
Morphine equivalents consumed in the PACU (mg kg^−1^)	0.06 (0.07)	0.09 (0.09)	0.03 (−0.02 to 0.08)	0.204
Aldrete score 15 min into PACU	9 (8–9)	9 (8–9)	0	0.613
Time spent in the PACU (min)	120 (45)	115 (44)	5 (−21 to 31)	0.711

All values are represented as mean (standard deviation), median (inter-quartile range), mean or actual difference (95% confidence interval).

CI, confidence interval

∗ Measured at 120 min into surgery.

† Time between administration of neuromuscular reversal agent and extubation.

**Fig. 3:**
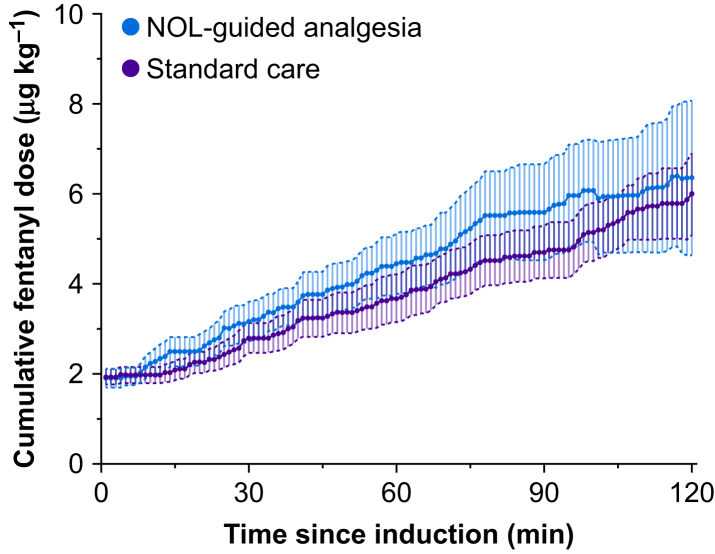
Cumulative fentanyl dosing in Nociception Level (NOL)-guided patients (red symbols) and patients receiving standard clinical care (blue symbols). Data are 1-min averages (95% confidence intervals).

Three subjects received remifentanil because of the inability of fentanyl to subdue hypertension (standard care group) or high NOL values (NOL-guided group). When remifentanil was given, no further fentanyl was administered. One subject, randomised to standard care, received an equivalent of fentanyl 8.5 μg kg^−1^ (remifentanil given from *t*=120–180 min surgery time). Two other subjects, randomised to NOL guidance, received an equivalent of fentanyl 14 μg kg^−1^ (t=90–210 min), and a fentanyl equivalent of 8 μg kg^−1^ (t=130–260 min), respectively. The cumulative administered fentanyl dose did not differ between treatment groups at 120 min into surgery with 6.4 (4.2) (NOL-guided group) *vs* 6.0 (2.2) μg kg^−1^ (standard care; mean difference=0.35 μg kg^−1^ with 95% CI −1.93–2.65 μg kg^−1^, *P*=0.749; Figure 3 and Table 2). Also, at *t*=60 (mean difference 0.77 μg kg^−1^ with 95% CI −0.24–1.78 μg kg^−1^) and 180 min (mean difference 4.15 μg kg^−1^ with 95% CI −2.8–11.1 μg kg^−1^) no differences in fentanyl dosing were observed. In terms of dose per kg, the variability was higher in the NOL-guided group: 66% *vs* 37%. The area-under-the curve (AUC) of the receiver operating characteristic curve was calculated to evaluate the relevance of the effect of the intervention (opioid dosing based on NOL *vs* standard care) on opioid dosing at 2 h into surgery and at the end of surgery. The AUC was not significantly different from 0.5 (random effect) at both time points: 2 h into surgery, AUC=0.51 with 95% CI 0.29–0.73, *P*=0.930, and at the end of surgery, AUC=0.56 with 95% CI 0.39–0.72, *P*=0.484. The number of doses and the average amount of fentanyl given per dose did not differ between groups. At the time of fentanyl dosing, MAP, systolic BP, and NOL did not differ between groups. However, HR was higher in the standard care group; 69 (13) against 79 (13) beats min^−1^ (mean difference −10 beats min^−1^ with 95% CI −17.4 to −2.61, *P*=0.009).

### Measurements made in the PACU

Cumulative morphine or piritramide consumption (in morphine equivalents) in the PACU did not differ between groups, albeit the absolute values show 30% less morphine use in the NOL-guided group (NOL-guided group 0.06 [0.07] mg kg^−1^ *vs* standard care 0.09 [0.09] mg kg^−1^, mean difference −0.03 mg kg^−1^, with 95% CI −0.08–0.02 mg kg^−1^, *P*=0.204).

In both groups the Aldrete score reached values >8 in the majority of patients within 15 min in the PACU. Nausea occurred in seven and nine patients in the NOL-guided and standard care groups, respectively. The time spent in the PACU was 115–120 min in the two treatment groups. No awareness was noted in the PACU or later on the ward.

### Blood hormone concentrations

**Fig. 4:**
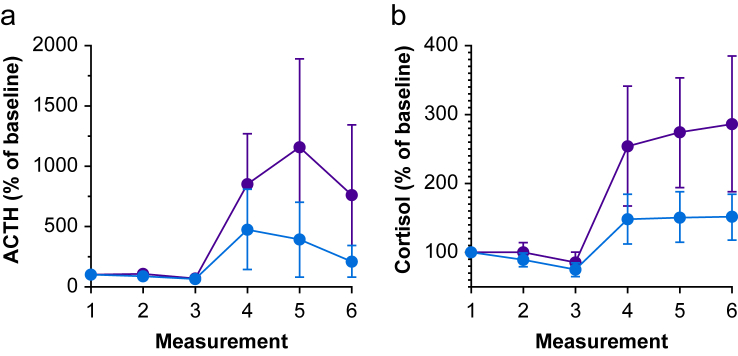
Adrenocorticotropic hormone (ACTH) (a) and cortisol (b) concentrations from induction on until discharge from the PACU in subjects receiving standard clinical care (red symbols) and subjects receiving fentanyl dosing based on the Nociception Level index (blue symbols). Measurements were (1) 10–30 min before induction, (2) 1–2 min after intubation, (3) 1–2 min after incision, (4) at skin closure, (5) 15 min into the PACU, and (6) at discharge from the PACU. Data are mean values relative to baseline (1) (95% confidence interval).

ACTH and cortisol concentrations at baseline (measurement 1) were 27 (15) and 23 (16) ng L^−1^ in NOL-guided and standard care groups, respectively, equivalent cortisol concentrations were 0.404 (0.157) and 0.312 (0.122) nmol L^−1^, respectively. Hormone concentrations relative to baseline levels are given in Figure 4. ACTH and cortisol were consistently low after intubation and skin incision, but increased in both groups during surgery, an effect that persisted in the PACU. Relative to baseline, the increase of ACTH and cortisol was 1.5–2-fold greater in the group that received standard care compared with those who received NOL index-guided fentanyl dosing (*P*<0.01).

### Simulation study

Estimated plasma fentanyl concentrations at the end of surgery were 1.05 (0.5) ng ml^−1^ in the NOL-guided group and 0.94 (0.3) ng ml^−1^ in the standard care group (mean difference 0.20 ng ml^−1^ with 95% CI −0.41–0.45 ng ml^−1^, *P*=0.102). Two hours after the end of surgery the plasma fentanyl concentrations decreased by 39 (7)% and 38 (9)% in the NOL-guided group and the standard care group, respectively (mean difference −0.02 with 95% CI −0.07–0.03, *P*=0.46).

## Discussion

We observed that multiparameter NOL index-guided opioid dosing rather than dosing based on haemodynamic indices (BP and HR) results in a 1.6-point reduction in postoperative pain scores. In addition, an important finding was that stress hormone concentrations during and after surgery were on average 50% lower in subjects who received opioid treatment based on their nociceptive state during surgery.

The majority of postoperative patients experience relatively high pain scores during recovery, which, apart from causing reduced patient wellbeing, is a source of preventable morbidity.^10^, ^11^, ^12^ Additionally, high pain scores in postoperative patients may have consequences with respect to longer term opioid consumption.^13^^,^^14^ To combat high levels of pain, physicians will prescribe more opioids at higher doses and prescriptions will often be extended beyond the hospital stay.^13^^,^^14^ Overprescribing may cause respiratory depression and additionally is one of the causes of the current opioid prescription epidemic worldwide. Moreover, high pain scores after surgery is one of the main factors associated with development of persistent pain.^22^ Hence, given all of the above, it is important to prevent high pain levels after surgery. As both opioid underdosing and overdosing during anaesthesia are associated with high postoperative pain scores, we designed a study in which we examined whether opioid dosing based on the individual nociceptive state of the patient during surgery, as measured by the NOL index, would improve postoperative pain scores.

While the result of our study was affirmative, we also show that the opioid dose given during surgery *per se* was not the determinant of reduced postoperative pain scores, as we observed similar cumulative fentanyl doses administered in the two treatment groups. In order to obtain an indication of the plasma fentanyl concentrations at the end of surgery, we performed a simulation study taking the individual fentanyl doses and timing of dosing during surgery into account. Estimated plasma concentrations were similar between treatment groups (NOL-guided group 1.05 ng ml^−1^ *vs* standard care group 0.94). However, we did observe a difference in the fentanyl dose variability between the two treatment groups (% coefficient of variation in dose per kg=66% in the NOL-guided group *vs* 37% in the standard care group, Figure 3), indicative of the more individualised and targeted dosing of opioids when the NOL index is used rather than haemodynamics. The variability in dosing was mirrored in the variability in estimated plasma concentrations (NOL-guided group 45% *vs* standard care group 0.94). The finding that nociception was more effectively treated is additionally grounded in the finding that stress hormones concentrations were lower in the NOL-guided group, both at the end of surgery and during PACU stay.

Currently, several nociception monitors are available for detection of nociceptive events during surgery.^5^ The algorithms used to derive the level of nociception differ among monitors and most monitors measure single physiological variables such as HR variability or combine two variables such as BP and HR. Two recent systematic reviews indicate that although some of these monitors show promising results, no definitive conclusions regarding their effect on anaesthesia-related outcomes can be drawn.^23^^,^^24^ In contrast to the other nociception monitors, the multiparameter NOL index that we used has an algorithm based on machine learning technology.^9^ Earlier studies indicate that the NOL index outperforms other indices of nociception including BP, HR, the pulse plethysmographic amplitude and the surgical pleth index (GE, Healthcare, Helsinki, Finland; an index that combines pulse beat interval and pulse wave amplitude), in discriminating noxious from non-noxious stimuli.^6^, ^7^, ^8^ Additionally, we recently showed that NOL-guided remifentanil dosing results in improved haemodynamic stability compared with standard care.^2^ The earlier findings and the current observation of reduced pain scores in patients receiving NOL-guided fentanyl dosing indicate the added value of a multiparameter monitor driven by an artificial intelligence algorithm based on advanced statistical and machine learning technologies.

We used absolute cut-offs to initiate fentanyl dosing (i.e. a NOL index value of 25 in the intervention arm and systolic BP of 140 mm Hg or HR of 90 beats min^−1^ in the standard care arm. One may argue that using haemodynamic cut-offs relative to presurgical baseline values would have been a more appropriate approach. We based our thresholds on previous outcome and validation studies with the NOL index.^2^^,^^6^ In a recent study on the effect of intraoperative hypotension and cardiac and renal outcome, Salsami and colleagues showed that associations based on relative thresholds were no stronger than those based on absolute cut-offs.^25^ A review of recently published RCTs on the use of nociception monitors during general anaesthesia (see Meijer and colleagues^23^ and references cited therein) show significant heterogeneity in the use of relative *vs* fixed thresholds in the comparator groups. Just seven of 15 studies use a relative BP threshold) whereas 14/15 use a fixed threshold in the intervention arm. This indicates the absence of agreement in the use of thresholds of comparator groups in nociception monitoring studies. In addition, it is questionable whether presurgical BPs are a valid reflection of the patients' ‘true’ BP and are useful during anesthesia.^26^^,^^27^ The use of relative thresholds seems most appropriate when patients differ considerably in their haemodynamic status. In our study all patients were quite comparable in this respect. None of the subjects had hypertension and BPs at screening and before surgery were similar with 95% of MAP values within the 92–102 mm Hg range. Hence, we contend that using a cut-off in systolic BP of 140 mm Hg did not disadvantage any of the subjects or treatment arms. We do appreciate, however, that different thresholds in the intervention and control arms might have resulted in a different outcome. Further studies are needed to determine the influence of different cut-offs on outcome and come to a general agreement on what cut-offs are optimal in studies on nociception.

### Limitations

First, in three subjects (two in the NOL-guided group), fentanyl was unable to sufficiently subdue hypertension or high nociception values. In these patients, continuous remifentanil was administered during the course of the surgical procedure. Given the rapid offset of remifentanil effect after the end of infusion, this may have impacted (increased) postoperative pain scores in these three patients. However, since the number of patients that received remifentanil was small and two of these subjects received NOL-guided care, we do not think that this influenced the outcome of our study. Second, both piritramide and morphine were allowed for treatment of postoperative pain. This was related to the fact that morphine is the opioid of choice in one centre (Leiden University Medical Centre) and piritramide in the other. As the distribution was similar between groups and the opioids were given in response to the pain score of the patient, we contend that this did not affect our study outcome, particularly since there was no effect of centre (and consequently of pain treatment) on postoperative pain scores. Third, the sample size was relatively small, but our intention was to detect a relatively large difference in postoperative pain scores that are clinically relevant. We observed a difference of 1.6 points (a 33% difference) on the 11-point NRS scale between groups with 84% of subjects who had received NOL-guided anaesthesia with pain scores of 4 or less *vs* 40% of subjects after standard care. These differences are clinically relevant,^19^ and were observed in both study centres, indicative that the value of the NOL index is generalisable across centres. Fourth, the primary outcome of our study was pain measurement in the first 90 min in the PACU. Although pain in the PACU is relevant and a determinant of later events, future studies should address long-term outcome such as persistent postoperative pain, 30-day complication rate, prolonged opioid use, and patient reported outcome.

## Conclusions

In this two-centre RCT, we observed that subjects undergoing elective abdominal surgery experience less pain when opioid dosing is guided by the multiparameter Nociception Level index compared with standard care. As opioid dosing did not differ between groups, we relate the difference in outcome to individualised dosing targeted at increased nociception. Future studies in larger more diverse populations, under uncontrolled conditions, should address the long-term outcome benefit of intraoperative analgesia guidance by the NOL index.

## Authors' contributions

Substantial contribution to conception and design: AD, MN, CM, LA, MB

Acquisition of data: FM, MH, TR, ST

Supervised the experimental part of the study: MB, CM, PC, AD, MN

Data analysis: EO, AD, MvV, MN

Interpretation of the data: all authors

Drafting of the manuscript/commenting on the manuscript (including revision): all authors

Approved the final version of the manuscript: all authors

All authors agree to be accountable for all aspects of the work and ensure that all questions related to the accuracy or integrity of any part of the work are appropriately investigated and resolved.

## Declarations of interest

AD received a speaker fee from Medasense. The Anesthesia & Pain Research Unit received/receives funding from MSD Nederland BV, Grünenthal GmbH (Germany), Bedrocan BV (Netherlands), AMO Pharma (UK), all unrelated to the current project. All other authors report no conflict of interest.

## Funding

Medasense Ltd (Israel), and from institutional and departmental sources.

## Figures and Tables

**Figs1:**