# Supplementary figures and images for: Reduced postoperative pain using Nociception Level-guided fentanyl dosing during sevoflurane anaesthesia: a randomised controlled trial
Source: Br J Anaesth. 2020 Sep 17;125(6):1070–8. doi: 10.1016/j.bja.2020.07.057 (PMC7771114; doi:10.1016/j.bja.2020.07.057)

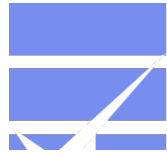

## CONSORT 2010 Flow Diagram

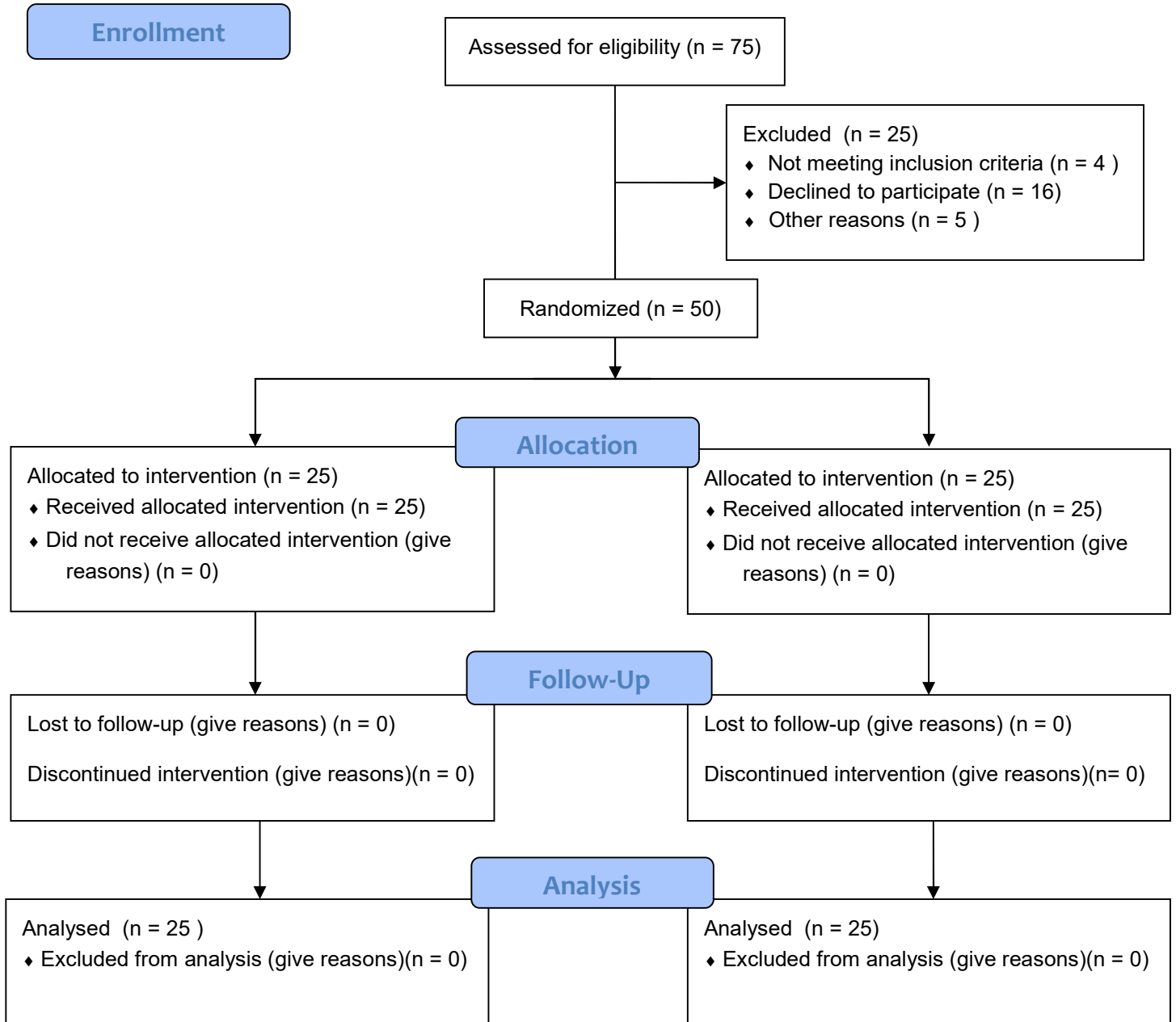

Supplement: Multimedia component 1 [file mmc1.pdf]
